# A cohort study of retinal detachment among Swedish construction workers

**DOI:** 10.5271/sjweh.4100

**Published:** 2023-10-01

**Authors:** Kevin D Schott, David Kriebel, Susan R Sama, Bryan O Buchholz, Bengt Järvholm, Jens Wahlström

**Affiliations:** 1 Department of Public Health, University of Massachusetts Lowell, Lowell, MA, USA.; 2 Department of Biomedical Engineering, University of Massachusetts Lowell, Lowell, MA, USA.; 3 Department of Public Health and Clinical Medicine, Umeå University, Umeå, Sweden.

**Keywords:** exertion, eye disease, manual labor, occupational disease, occupational exposure, Sweden

## Abstract

**Objective:**

Retinal detachment (RD) has been associated with exposure to heavy lifting. Many occupations within the construction industry are likely to involve lifting tasks. We investigated the association between occupational heavy lifting and rhegmatogenous RD in a retrospective cohort study of Swedish construction workers.

**Methods:**

We studied Swedish construction workers who participated in an industry-wide health and safety program from 1971 to 1993. Individual occupation codes were linked to a job exposure matrix, assigning intensity of exposure to heavy lifting to each worker. The Swedish National Patient Register was used to identify cases of RD that occurred during follow-up through the end of 2012. We used Poisson regression modeling to calculate incidence rates of RD associated with heavy lifting, age and other covariates. A subcohort of those age ≤25 years at enrollment was studied to reduce bias from missing exposure information from work prior to enrollment.

**Results:**

Of 256 241 construction workers, 17% were classified with high exposure to heavy lifting in their occupation. Within the cohort, 1588 cases of RD were identified. Average exposure intensity of heavy lifting was not associated with risk of RD. However, RD risk increased with increasing cumulative exposure to heavy lifting, both in the full cohort and subcohort of those who were ≤25 years old at entry into the construction-worker cohort.

**Conclusion:**

Construction workers’ risk of RD appeared to increase with time spent exposed to heavy lifting.

Retinal detachment (RD) is a major cause of visual impairment that can lead to blindness if not treated promptly. General population incidence rates (IR) range from 8 to 18 cases per 100 000 with most occurring at 60–70 years of age (1–5). The most common form, called rhegmatogenous retinal detachment, typically occurs in conjunction with posterior vitreous detachment, a condition in which the cortex of the vitreous humor pulls away from the retinal surface. This separation often occurs without incident among older adults. However, if this separation does not occur properly, tears or holes may occur in the retina, leading to interruption in blood flow and retinal cell death. If surgical intervention is not undertaken quickly, permanent loss of vision may occur (6). The risk of RD rises steeply with age, peaking near the sixth decade and declining thereafter (2).

Several studies have reported that heavy lifting may lead to an increased risk of RD. Mattioli et al (7, 8) first reported an increased risk of RD associated with lifetime cumulative heavy lifting in an Italian case-control study. A second case–control study by Kriebel et al (9) supported Mattioli’s finding. Three population-based cohort studies have yielded somewhat inconsistent results (10–12). Curti et al (10) studied rates of hospitalization for RD in the Italian region of Tuscany and found higher rates of RD among men with manual compared to non-manual jobs. In contrast, a population register-based study of Danish men found the reverse: the RD incidence was lower among those with jobs classified as manual labor compared to non-manual jobs (11). Neither of these two last studies controlled for important potential confounders including myopia and social class. A third cohort study has also been published using data on a cohort of Swedish men identified at their conscription into military service (12). This study, which benefited from detailed confounder information and a job exposure matrix (JEM) to estimate participants’ exposure to heavy lifting, found a positive association between increased RD risk and jobs involving heavy lifting.

The objective of the current study was to investigate the hypothesis that heavy lifting increases the risk of RD by analyzing data from a large cohort of construction workers collected during the Swedish Construction Industry’s Organization for Working Environment, Occupational Health, and Safety (Bygghälsan) surveillance program established in 1968 (13). Numerous occupational health studies have been published using this data set (13–17). Because construction work often involves heavy physical labor, this cohort provided a good opportunity to further investigate the heavy lifting–RD hypothesis.

## Methods

### Data

A cohort of male Swedish construction workers was followed over a 26-year period for the occurrence of RD. The open longitudinal follow-up study was conducted among construction workers who participated in the Bygghälsan program. The full cohort consisted of 389 132 Swedish construction workers who participated in at least one health survey between 1971 and 1993. Participation was voluntary and ≥80% of eligible workers completed one or more health examinations during this observation period (14). Workers entering the study were given a health examination by a nurse, and 0–3 additional health examinations were administered while in the study. At each exam, health data such as height, weight, and blood pressure were measured, and general work- and health-related questions were asked such as job title and smoking habits. Ophthalmological examinations were not given at any health examination.

Two different versions of a health questionnaire were fielded during health examinations, one administered between 1971 and 1974 and one between 1989 and 1993. These surveys provided self-reported information on chronic health conditions and health behaviors, work environment hazards, ergonomic factors encountered at work, and contact with chemicals and other potentially harmful agents.

The Swedish National Patient Register was used to identify cases of RD that occurred among the Bygghälsan participants, as well as deaths from any cause. Health outcome data were available from 1987 through 2012, allowing for a maximum of 26 years of follow-up. The linked data set contained information from one or two health examinations per worker during the observation period.

### Inclusion and exclusion

Workers aged 16–60 years at their first health examination were selected from the available data to form the study cohort. Each worker was followed from entry into the study until an occurrence of RD, death, emigration, or end of follow-up on 31 December 2012. Health data prior to entry into the Bygghälsan program was not available for this study, and therefore the status of previous RD was unknown.

**Figure 1:**
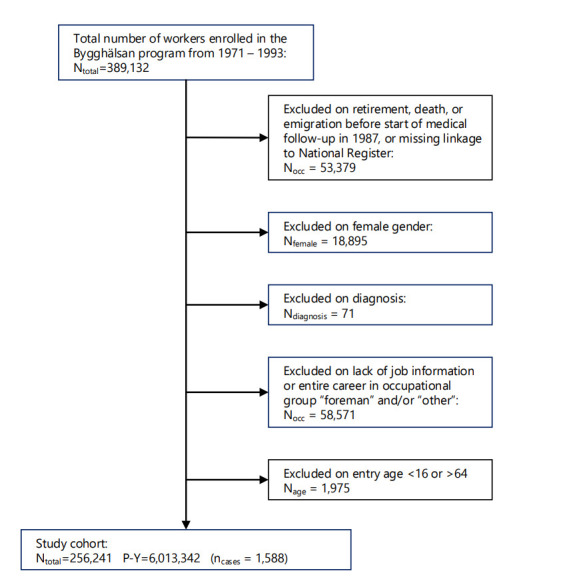
Study inclusion and exclusion.

Female workers were excluded from the study as their representation was very low (<6%) and there were insufficient numbers of RD events to study. Workers with no job titles or those that fell into the “other” occupation group were excluded, as were foremen because their work tasks were not evaluated and many of them probably had careers as construction workers prior to becoming foremen (figure 1) (15).

### Outcome definition

An RD case was defined as a participant with “retinal detachment and breaks” per International Classification of Disease (ICD) diagnosis codes 361.0* (ICD-9) or H33.0* (ICD-10). Up to eight additional diagnosis codes were contemporaneous with the RD diagnosis. If any of these additional diagnosis codes indicated trauma, diabetes, or other non-idiopathic causes of the detachment, the participant was excluded from the study.

### Exposure assessment

Workers reported their construction job titles at health examinations using codes that were standard in the industry at the time. These codes varied over time, but were combined by occupational hygienists into 22 occupational groups performing similar work tasks and having similar training. An “other” work code was assigned to those whose job titles could not be confidently assigned to a known category (table 1).

**Table 1:** Occupations and expert ratings of average levels of heavy lifting in the Swedish construction cohort (N=256 241, total person-years (PY)=6 013 342). [JEM=job exposure matrix; 1=low, 2=medium, 3=high.]

Occupational group		N	%	PY	% in JEM ^a^
JEM level 1					10.4
	Salaried employees	13 452	5.25	307 695	
	Machine operators	10 006	3.90	232 912	
	Crane operators	3134	1.22	71 439	
JEM level 2					73.4
	Asphalt workers	3035	1.18	68 857	
	Floor layers	5310	2.07	125 141	
	Drivers	4018	1.57	92 385	
	Glaziers	2601	1.02	62 028	
	Insulators	2601	1.02	60 951	
	Refrigeration repairers	1302	0.51	30 668	
	Plumbers and pipe fitters	22 896	8.94	540 577	
	Painters	21 789	8.5	519 086	
	Sheet metal workers	11 648	4.55	277 135	
	Electricians	35 578	13.88	857 709	
	Repairers	2753	1.07	63 215	
	Earthmoving workers	10 644	4.15	241 111	
	Woodworkers	63 887	24.93	1 520 312	
JEM level 3					16.2
	Rock Workers	2613	1.02	56 539	
	Concrete workers	28 935	11.29	653 239	
	Bricklayers	8710	3.4	201 445	
	Pasteboard layers (roofers)	1329	0.52	30 898	

^a^ JEM links occupational groups to average levels of heavy lifting assigned by occupational hygienists (see text).

Exposure to occupationally-related heavy lifting was assessed using a JEM approach. During the Bygghälsan surveillance program, occupational hygienists conducted on site assessments of jobs within each of these groups, grading various work environment exposures. The assessment information was then used to establish exposure intensity for each occupational group (15–17). Heavy lifting exposure focused on the degree of back loading, providing an indication of full-body involvement in the lifting activity. One of three levels of exposure intensity (1=low, 2=medium, 3=high) was assigned to each occupational group.

Job title was identified at health examinations. If a worker participated in only one health examination, the worker was assumed to remain in the same occupational group for the entire length of the study. If a change in occupational groups was identified between two health examinations, the worker was assumed to be in their initial occupational group until their second examination date, and in their second group for all years after their second examination. In the absence of death or emigration information, continued participation as a construction worker in the last identified occupational group was assumed until retirement age of 65. The official age for governmental pension was 65 years until 2003.

### Covariates

Height, weight, and blood pressure were measured at health examinations. The available data contained only height and weight data for the entrance examination and, therefore, the calculated body mass index (BMI) necessarily assumes that neither height nor weight changed during the study. Ranges published by the World Health Organization (18) were used to define four BMI levels (underweight, normal, overweight, obese). The extreme groups had low frequencies (<5%) and therefore “underweight” and “obese” were combined with “normal” and “overweight”, respectively.

The hypertension categorical variable was constructed based on the last available blood pressure measurement. Two levels of blood pressure (normal, hypertension) were defined corresponding to systolic and diastolic classifications in the 2013 European Society of Hypertension guidelines for arterial hypertension (19).

A single smoking status categorical variable was constructed using the levels from the examination (non-, former, moderate, heavy) with each study participant receiving the highest value from their available data. Roughly 7% of participants had no smoking status information, and 51% only had smoking status recorded from one examination. Only about 6% of participants reported their smoking status lower (ie, less smoking per day) during their subsequent examination than on their first. The smoking status categorical variable was initially comprised of four levels – non-, former-, moderate-, and heavy smoker – however no information was available as to when a smoker stopped smoking or to what degree they smoked prior to becoming a former smoker. The smoking status categorical was therefore revised to two levels: ever- versus never-smokers.

Age and myopia are strong risk factors for RD. Age was categorized into eight levels: <45, 5-year increments from 45–75, and >75 years of age. Unfortunately, no direct measure of myopia was available as ophthalmologic assessments were not part of the health examinations. From 1971–1974, the health questionnaire contained the question: “Do you have to use glasses all of the time?” Responses were recorded as “yes” or “no, or not answered”. Although myopia is not the only reason eyeglasses may be worn “all of the time”, it is one of the most common reasons (20), and so we used this question response as a proxy for myopia.

### Statistical analysis

Person-years were calculated from the start of follow-up in 1987 or entrance into the program until an RD case or censoring due to death, emigration, or the end of study (31 December 2012). Poisson regression analysis via generalized linear models was used to calculate IR and IR ratios (IRR). All analyses were performed using RD status as the dependent count variable and person-years as the offset variable. As age is a well-known factor for RD, all analyses except those identified as “crude” were age-adjusted to the age distribution of the cohort at the end of follow-up in 2012.

Average intensity of lifting, cumulative lifting, and duration of construction work were used as measures of exposure. For each year, the exposure intensity provided by the JEM (table 1) was used as the average for that year. Cumulative exposure (CE) was calculated as the sum of the annual average intensities during working years (≤65 years or until job transfer to foreman or “other” occupation). Participants’ individual CE scores were grouped into quintiles for analysis. Duration of exposure was calculated as the sum of working years and also grouped into quintiles.

All calculations were performed using SAS 9.4 (SAS Institute, Cary. NC, USA) with two-sided P<0.05 as the threshold for assessing significance. Procedure GENMOD (generalized linear model) was used to execute regressions, specifying a log link function and Poisson distribution.

### Subgroup analyses

Many members of the cohort were employed prior to entry into the cohort and nothing is known about their work at that time. As a result, exposure histories are incomplete for most cohort members. For the study cohort, the mean age at entry into the Bygghälsan surveillance program was 30 (range 16–59) years (table 2).

**Table 2:** Swedish construction cohort characteristics. [RD=retinal detachment; IR=incidence rate; PY=person years; SD=standard deviation]

Characteristic		Full cohort N=256 241 RD cases=1588 PY=6 013 342 Crude IR=36.4			≤25 years at entry N=103 883 RD cases=373 PY=2 563 016 Crude IR=14.6	
		Mean (SD)	Range		Mean (SD)	Range
Age when entering the Bygghälsan program ^a^		29.7 (9.5)	16–59		21.2 (2.4)	16–25
	Age when entering follow-up	38.5 (12.6)	16–64		27.4 (6.2)	16–41
	Number of years of follow-up	24.1 (5.0)	1–26		25.4 (3.0)	1–26
	Age when RD occurred	60.2 (10.8)	24–89		49.8 (9.0)	24–64

^a^ A subcohort of those age ≤25 at entry into the Bygghälsan program was defined to minimize exposure histories prior to enrollment. ^b^ Follow was between 1987 and 2013

To address this problem, a subgroup of the full study cohort was constructed consisting of workers entering the Bygghälsan surveillance program at or before 25 years of age, thereby minimizing pre-study time available for unknown exposure. Because the total duration of the study, from year of first entry (1987) until end of follow-up (2012) was 26 years, the period of follow-up for this subgroup was coincidentally limited to a study exit age of 67, and thus 98.7% of the ≤25-years subcohort was within normal working ages (up to age 65) for the entire study.

## Results

The final study population consisted of 256 241 male construction workers who contributed 6 013 342 person years of observation and experienced 1588 RD for a crude IR of 26.4 per 100 000 person years (table 2). The subcohort who were ≤25 years of age at entry, and therefore were likely to have more complete exposure histories, totaled 103 883 and contributed 43% of the person years and 23% of the RD cases.

As expected, the strongest risk factor for RD was age (table 3). Those who were overweight, with high blood pressure or were smokers had modestly increased risks of RD in crude analyses. However, after controlling for age, each of these associations became essentially null, and so these covariates were not included in the models investigating occupational risks. In contrast, those who reported using glasses all the time were about 2.7 times more likely to have an RD, and this association was unaffected by age adjustment. This analysis however was limited to the approximately 31% of the cohort who answered this survey question between 1971 and 1975.

**Table 3:** Participant characteristics among full 278 409 Swedish construction worker cohort; univariate analysis of potential retinal detachment (RD) risk factors. [IR=incidence rate per 100 000 person years (PY); IRR=IR ratio; CI=confidence interval; BMI=body mass index.]

Covariate		PY	Cases	IR	IRR (95% CI)	Age-adjusted IRR (95% CI)
Age						
	≤45	2 432 874	155	6.4	1.00	
	46–50	779 344	128	16.4	2.58 (2.04–3.26)	
	51–55	727 438	257	35.3	5.55 (4.54–6.77)	
	56–60	659 867	294	44.6	6.99 (5.76–8.50)	
	61–65	563 360	306	54.3	8.53 (7.03–10.34)	
	66–70	400 329	200	50.0	7.84 (6.36–9.67)	
	71–75	243 395	133	54.6	8.58 (6.80–10.81)	
	>75	195 028	98	50.3	7.89 (6.12–10.16)	
BMI						
	Normal	4 232 903	1 056	24.9	1.00	1.00
	Over	1 768 732	515	29.1	1.17 (1.05–1.30)	0.88 (0.79–0.98)
Blood pressure						
	Normal	3 994 595	953	23.9	1.00	1.00
	High	2 004 988	618	30.8	1.28 (1.16–1.41)	0.91 (0.82–1.01)
Smoking						
	Never	2 542 521	567	22.3	1.00	1.00
	Current or past	3 166 617	911	28.8	1.28 (1.15–1.42)	0.91 (0.82–1.01)
Uses glasses all the time						
	No	1 719 450	559	32.5	1.00	1.00
	Yes	137 032	120	87.6	2.69 (2.21–3.28)	2.67 (2.19–3.25)

### Occupational exposures

Before applying the JEM information on heavy lifting by occupation, a simple stratification of RD risk among those in manual versus non-manual jobs was conducted. The only non-manual workers in the Bygghälsan cohort were a fairly small group (5%) of salaried workers who were also employed in the construction industry. The manual versus non-manual comparison has been reported in three previous cohorts (10–12). In the present study, there was a nearly 50% *higher* age-adjusted IR of RD among non-manual versus manual workers (52.3/100 000 versus 35.8/100 000). Two previous studies reported a very similar result (11, 12). This counter-intuitive pattern may be due to uncontrolled confounding by myopia and higher social class, both of which are more prevalent in non-manual occupations and strong risk factors for RD (7–9, 12).

Further analyses of occupational heavy lifting were limited to the 103 883 construction workers who enrolled in the Bygghälsan program at ≤25 years (table 2) to limit the potential bias from missing work information for those who were older at enrollment. The simplest heavy lifting exposure measure was the average-intensity of exposure to heavy lifting provided by the JEM, scored as low, medium or high. This measure was only weakly associated with age-adjusted RD risk (supplementary material, www.sjweh.fi/article/4100, table S1).

In contrast, CE to heavy lifting was positively associated with RD risk (table 4). The CE metric represents the working lifetime sum of the annual heavy lifting intensity scores: 1, 2 or 3. So, for example a worker with a CE of 40, falling into the fourth quintile of CE, might have worked for 40 years in a low exposure job, or 20 years in a medium exposure job, or 13 years in a high exposure job, etc.

**Table 4:** Age-adjusted incidence rate (IR) ratios (IRR) of retinal detachment among Swedish construction workers classified by cumulative exposure to heavy lifting exposure (see text) and duration of construction work, for the subcohort ≤25 years old at entry into the Bygghälsan program. Exposure categories are quintiles and corresponding year ranges, eg, Q1=lowest 20%, consisting of 0–10 years of exposure, etc. [CI=confidence interval; PY=person years]

Exposure categories	IRR (95% CI)	Crude IR	Cases	PY
Q1: 0–10	1.00	5.9	34	574 586
Q2: 11–10	1.33 (0.84-2.13)	7.5	37	496 354
Q3: 21–10	1.56 (1.01-2.41)	11.2	54	481 527
Q4: 31–42	1.74 (1.15-2.62)	18.4	99	536 822
Q5: >42	1.75 (1.15-2.65)	31.4	149	473 900

The CE RD risk results in table 4 were not controlled for eye glass wearing because there were not enough participants who were given the version of the health questionnaire that included the eye glass question and who were ≤25 years at enrollment. When the eye glass variable and cumulative heavy lifting exposure were included in a Poisson regression model applied to the data available in the full cohort, there was no evidence of confounding (data not shown).

The CE metric has two components: the intensity of exposure and its duration. As noted, heavy lifting exposure intensity was only weakly associated with RD risk (supplementary table S1), but in contrast, duration of construction employment was associated with RD risk, although in a less clearly monotonic pattern than CE (supplementary table S2).

## Discussion

Mattioli and colleagues (7) first reported the finding of an increased risk of RD among those engaged in heavy physical labor. Subsequently there have been additional studies investigating this hypothesis, using various methods and source populations (8–12, 21). All but one (11) of the studies support the basic hypothesis that heavy lifting increases RD risk. We hypothesized that the Bygghälsan construction cohort would provide a valuable opportunity to further investigate the role of heavy lifting in RD.

In this large sample of Swedish construction workers, an increased risk of RD was observed among those with the highest CE to occupational heavy lifting. CE estimates account not only for intensity and frequency of lifting within each year but also the duration of time a worker is in the job. Duration of construction work alone was also associated with RD risk. The occupational groups with the highest risk of RD were rock workers (drilling and blasting for road construction), concrete workers, brick layers, and roofers (table 1).

A previous study of a large sample of the entire Swedish male population also found an association between heavy lifting and RD risk (12). Because the Farioli study used a cohort of military conscripts, detailed medical data were available, including myopia from an ophthalmologic exam at age 18. It is notable that non-manual workers also showed a higher RD risk than manual workers among the Swedish conscripts, but this difference was eliminated after controlling for the strong confounding effect of myopia.

Curti et al (11) used a JEM approach with a population-based registry study of the general working population of Danish men 20–59 years of age. RD was assessed for exposure based on annual intensity of heavy frequent lifting for 15 years of follow-up. This registry-based study included no information on myopia, and it is likely that the finding of higher RD risk among non-manual workers was due to uncontrolled confounding by a higher prevalence of myopia in the “unexposed to heavy lifting” group. Our study focused specifically on construction workers who were presumably more frequently exposed to heavy lifting than either the Danish or Swedish cohorts of the general population.

The large size of this prospective cohort study benefited from the high participation rate of workers in the Bygghälsan program and linkage to the essentially complete medical information from the Swedish national registries (13–17). Exposure assessments were conducted by occupational hygienists visiting work sites across different regions of Sweden early in the surveillance program and their results were reviewed and checked by multiple researchers. Because these assessments were conducted at the job level rather than for individuals, they were of course blind to outcome status. At the same time, there is necessarily some misclassification of individual exposures since jobs, not individual participants were evaluated. This misclassification of exposure is expected to be non-differential.

As with any JEM-based exposure assessment, social and personal aspects of the job performance that may lead to non-uniform exposure within the occupational group cannot be measured. For example, younger workers may do more of the harder work, either by volunteer or by social convention. We also had no knowledge of prior or non-occupational exposures.

A limitation of our study is a lack of detailed covariate data. Full assessment of risk factors such as BMI, hypertension, and smoking was not possible as few measurements were available for each participant across the entire duration of the study. These factors may change considerably across a person’s lifetime.

Myopia has been identified as a risk factor for RD (22, 23), however no direct measure was available. In this study, the variable “using glasses all the time”, a limited proxy for myopia, did not appear to confound the association with heavy lifting. Additionally, prior medical diagnoses were not available and therefore any previous eye trauma, disease, or RD are unknown.

The Bygghälsan was chosen as a potentially valuable resource for investigating the heavy lifting – RD hypothesis because of its large size, long follow-up and particularly because of the high prevalence of heavy lifting exposures in construction work. But a corollary of the frequent high exposure may be also a relatively low percentage of persons with low exposures. This may possibly explain the weak association between RD risk and the intensity of exposure (supplementary table S1). It may be that within the Bygghälsan cohort, the more important source of variability in exposure is the duration of employment rather than the particular demands of one construction job compared to another. Thus, CE (and duration) appears to be associated with RD risk, rather than a worker’s average level or intensity of heavy lifting. The cohort includes a small white-collar group of salaried workers who would have had low exposure to heavy lifting, but their usefulness as a comparison population for this study is compromised by the problem of confounding by myopia, which tends to be more prevalent among those holding non-manual jobs (12). Because this study lacks a good measure of myopia, we were unable to fully adjust for this possible source of bias.

One hypothesized biomechanical mechanism for increased RD with heavy lifting is the deformation of eye shape that may occur due to increased intraocular pressure when lifting against a closed airway. Performing strong muscular contractions with a closed airway, known as the Valsalva maneuver, is sometimes used in sport weightlifting specifically when lifting extremely heavy weights as the accompanying increase in intra-abdominal pressure is thought to provide stability to the spine (24, 25). In population-based studies of heavy lifting, it is not possible to know the frequency and degree which the Valsalva maneuver was used when lifting.

The results of this study support an association between cumulative occupational heavy lifting and RD. Many questions remain, however, about the magnitude of risk from particular levels and durations of exposure, and therefore effective prevention strategies.

## Supplementary material

Supplementary material
