# Supplementary material for: A cohort study of retinal detachment among Swedish construction workers
Source: Scand J Work Environ Health. 2023 Sep 29;49(7):518–25. doi: 10.5271/sjweh.4100 (PMC10837844; doi:10.5271/sjweh.4100)

A cohort study of retinal detachment among Swedish construction workers[1]

by Kevin D Schott, ScD,[2] David Kriebel, ScD, Susan R Sama, ScD, Bryan O Buchholz, PhD, Bengt Järvholm, PhD, Jens Wahlström, PhD

1. Supplementary tables
2. Correspondence to: Kevin D. Schott, University of Massachusetts, Lowell, Lowell, MA, USA. [E-mail: kevin.schott@carelon.com]

Table S1. Age adjusted incidence rate ratios (IRR) of RD among Swedish construction workers classified by average level of lifting exposure (see text), subcohort aged 25 and younger at entry into the Bygghälsan program. [IRR = incidence rate ratio; CI = confidence interval.]

Heavy Lifting	IRR (95% CI)
Low	1.00
Medium	1.04 [0.65-1.68]
High	1.15 [0.67-1.97]

Table S2. Age-adjusted incidence rate ratios of retinal detachment among Swedish construction workers classified by duration of construction work, for the subcohort ≤ 25 years old at entry into the Bygghälsan program. [IRR = incidence rate ratio; 95% CI = confidence interval

Quintiles of duration of construction work	Range of duration of employment (years)	IRR (95% CI)
Q1	0 – 5	1.00
Q2	6 – 11	1.06 [0.65-1.72]
Q3	12 – 16	1.76 [1.14-2.72]
Q4	17 – 21	1.94 [1.28-2.95]
Q5	> 21	1.61 [1.05-2.46]

Table 1. Occupations and expert ratings of average levels of heavy lifting in the Swedish construction cohort (1 = low, 2 = medium, 3 = high), total N = 256 241, total Person-Years = 6 013 342

Occupational group	N	%	Person-Years	% in JEM[a]
JEM level 1				10.4
Salaried employees	13 452	5.25	307 695	
Machine operators	10 006	3.90	232 912	
Crane operators	3 134	1.22	71 439	
JEM level 2				73.4
Asphalt workers	3 035	1.18	68 857	
Floor layers	5 310	2.07	125 141	
Drivers	4 018	1.57	92 385	
Glaziers	2 601	1.02	62 028	
Insulators	2 601	1.02	60 951	
Refrigeration repairers	1 302	0.51	30 668	
Plumbers and pipe fitters	22 896	8.94	540 577	
Painters	21 789	8.5	519 086	
Sheet metal workers	11 648	4.55	277 135	
Electricians	35 578	13.88	857 709	
Repairers	2 753	1.07	63 215	
Earthmoving workers	10 644	4.15	241 111	
Woodworkers	63 887	24.93	1 520 312	
JEM level 3				16.2
Rock Workers	2 613	1.02	56 539	
Concrete workers	28 935	11.29	653 239	

Bricklayers	8 710	3.4	201 445
Pasteboard layers (roofers)	1 329	0.52	30 898

[a] JEM: job exposure matrix linking occupational groups to average levels of heavy lifting assigned by occupational hygienists (see text).

Table 2. Swedish construction cohort characteristics. A subcohort of those age ≤25 at entry into the Bygghälsan program was defined to minimize exposure histories prior to enrollment. [RD = retinal detachment.]

	**Full cohort**		**Under 25 at entry**	
**Characteristic**	**Mean (SD)**	**Min-Max**	**Mean (SD)**	**Min-Max**
Age when entering the Bygghälsan program	29.7 (9.5)	16-59	21.2 (2.4)	16-25
Age when entering follow-up	38.5 (12.6)	16-64	27.4 (6.2)	16-41
Number of years of follow-up (1987-2013)	24.1 (5.0)	1-26	25.4 (3.0)	1-26
Age when RD occurred	60.2 (10.8)	24-89	49.8 (9.0)	24-64
Number participants	256 241		103 883	
RD Cases	1 588		373	
Person Years	6 013 342		2 563 016	
Crude incidence rate per 100 000 person	26.4		14.6	

Table 3. Participant characteristics among full 278 409 Swedish construction worker cohort; univariate analysis of potential RD risk factors. [RD = retinal detachment; IR = incidence rate per 100 000 P-Y; IRR = incidence rate ratio; CI = 95% confidence interval; BMI = body mass index.]

**Covariate Age**	**Person Years**	**Cases**	**IR**	**IRR [CI]**	**Age-adjusted IRR [CI]**
<=45	2 432 874	155	6.4	1.	
46 – 50	779 344	128	16.4	2.58 [2.04 - 3.26]	
51 – 55	727 438	257	35.3	5.55 [4.54 - 6.77]	
56 – 60	659 867	294	44.6	6.99 [5.76 - 8.50]	
61 – 65	563 360	306	54.3	8.53 [7.03 - 10.34]	
66 – 70	400 329	200	50.0	7.84 [6.36 - 9.67]	
71 – 75	243 395	133	54.6	8.58 [6.80 - 10.81]	
>75	195 028	98	50.3	7.89 [6.12 - 10.16]	
**BMI**					
Normal	4 232 903	1 056	24.9	1.	1.
Over	1 768 732	515	29.1	1.17 [1.05 - 1.30]	0.88 [0.79-0.98]
**Blood pressure**					
Normal	3 994 595	953	23.9	1.	1.
High	2 004 988	618	30.8	1.28 [1.16 - 1.41]	0.91 [0.82-1.01]
**Smoking**					
Never	2 542 521	567	22.3	1.	1.
Current or past	3 166 617	911	28.8	1.28 [1.15 - 1.42]	0.91 [0.82-1.01]
**Uses glasses all the time**					
No	1 719 450	559	32.5	1.	1.
Yes	137 032	120	87.6	2.69 [2.21 - 3.28]	2.67 [2.19-3.25]

Table 4. Age-adjusted incidence rate ratios (IRR) of retinal detachment among Swedish construction workers classified by cumulative exposure to heavy lifting exposure (see text) and duration of construction work, for the subcohort ≤ 25 years old at entry into the Bygghälsan program. [IRR = incidence rate ratio, 95% CI = 95% confidence interval, Exposure categories = quintiles and corresponding year ranges, e.g. Q1 = lowest 20%, consisting of 0-10 years of exposure, etc.]

**Exposure categories**		**IRR [CI]**	**Crude IR**	**Cases**	**Person Years**
Q1:	0 - 10	1.	5.9	34	574 586
Q2:	11 - 10	1.33 [0.84-2.13]	7.5	37	496 354
Q3:	21 - 10	1.56 [1.01-2.41]	11.2	54	481 527
Q4:	31 - 42	1.74 [1.15-2.62]	18.4	99	536 822
Q5:	> 42	1.75 [1.15-2.65]	31.4	149	473 900

Figure 1. Study inclusion and exclusion

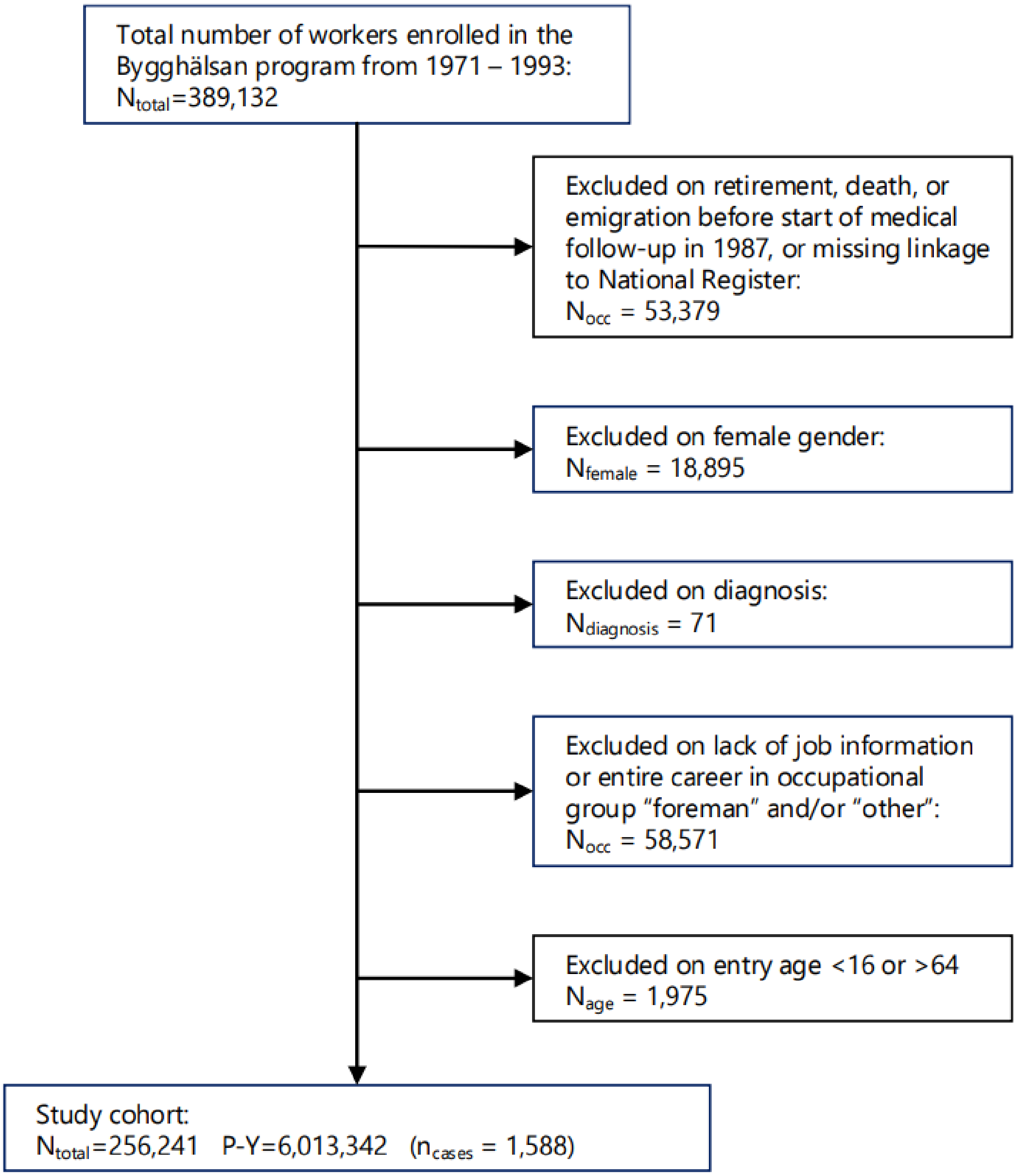

Supplement: Supplementary material [file SJWEH-49-518-S001.pdf]
